# Supplementary material for: Coagulation disorders in patients with severe hemophagocytic lymphohistiocytosis
Source: PLoS One. 2021 Aug 3;16(8):e0251216. doi: 10.1371/journal.pone.0251216 (PMC8330932; doi:10.1371/journal.pone.0251216)
Supplement: S1 Table — (DOCX) [file pone.0251216.s001.docx]

**S1 Table: HLH 2004 criteria (adapted from Henter et al, Pediatr Blood Cancer 2007)**

| The diagnosis of HLH can be established if one of either 1 or 2 below is fulfilled  (1) A molecular diagnosis consistent with HLH  (2) Diagnostic criteria for HLH fulfilled (five out of the eight criteria below)  (A) Initial diagnostic criteria (to be evaluated in all patients with HLH)  Fever  Splenomegaly  Cytopenias (affecting ≥ 2 of 3 lineages in the peripheral blood)  Hemoglobin < 90 g/L (in infants < 4 weeks: hemoglobin < 100 g/L)  Platelets < 100 x 10^9^/L  Neutrophils < 1.0 x 10^9^/L  Hypertriglyceridemia and/or hypofibrinogenemia:  Fasting triglycerides ≥ 3.0 mmol/L (i.e., ≥ 265 mg/dL)  Fibrinogen ≤ 1.5 g/L  Hemophagocytosis in bone marrow or spleen or lymph nodes  No evidence of malignancy  (B) New diagnostic criteria  Low or absent NK-cell activity (according to local laboratory reference)  Ferritin ≥ 500 mg/L  Soluble CD25 (i.e., soluble IL-2 receptor) ≥ 2400 U/ml |
| --- |
|  |
