# Supplementary material for: Coagulation disorders in patients with severe hemophagocytic lymphohistiocytosis
Source: PLoS One. 2021 Aug 3;16(8):e0251216. doi: 10.1371/journal.pone.0251216 (PMC8330932; doi:10.1371/journal.pone.0251216)
Supplement: S2 Table — (DOCX) [file pone.0251216.s002.docx]

**S2 Table: HScore** (adapted from Fardet et al, Arthritis Rheumatol 2014)

| **Known underlying immunosuppression**  (HIV or immunosuppressive treatment)  **Temperature**  **Number of cytopenia**  Leucopenia < 5 G/L  Platelets < 110 G/L  Hb < 9∙2 g/dL  **Ferritin (ng/mL)**  **Triglyceride (mmol/L)**  **Fibrinogen (g/L)**  **Serum glutamic oxaloacetic**  **transaminase (UI/L)**  **Hemophagocytosis features on bone**  **marrow aspiration** | 0 (no) or 18 (yes)  0 (< 38∙4°C), 33 (38∙4 - 39∙4°C)  49 (>39∙4°C)  0 (1 lineage), 24 (2 lineages)  or 34 (3 lineages)  0 ( <2000), 35 (2000 – 6000)  or 50 (>6000)  0 (>1∙5), 44 (1∙5 – 4) or 64 (>4)  0 if >2∙5g/L  30 if ≤2∙5g/L  0 (<30) or 19 (≥30)  0 (no) or 35 (yes) |
| --- | --- |
|  |  |
