# Supplementary figures and images for: Coagulation disorders in patients with severe hemophagocytic lymphohistiocytosis
Source: PLoS One. 2021 Aug 3;16(8):e0251216. doi: 10.1371/journal.pone.0251216 (PMC8330932; doi:10.1371/journal.pone.0251216)

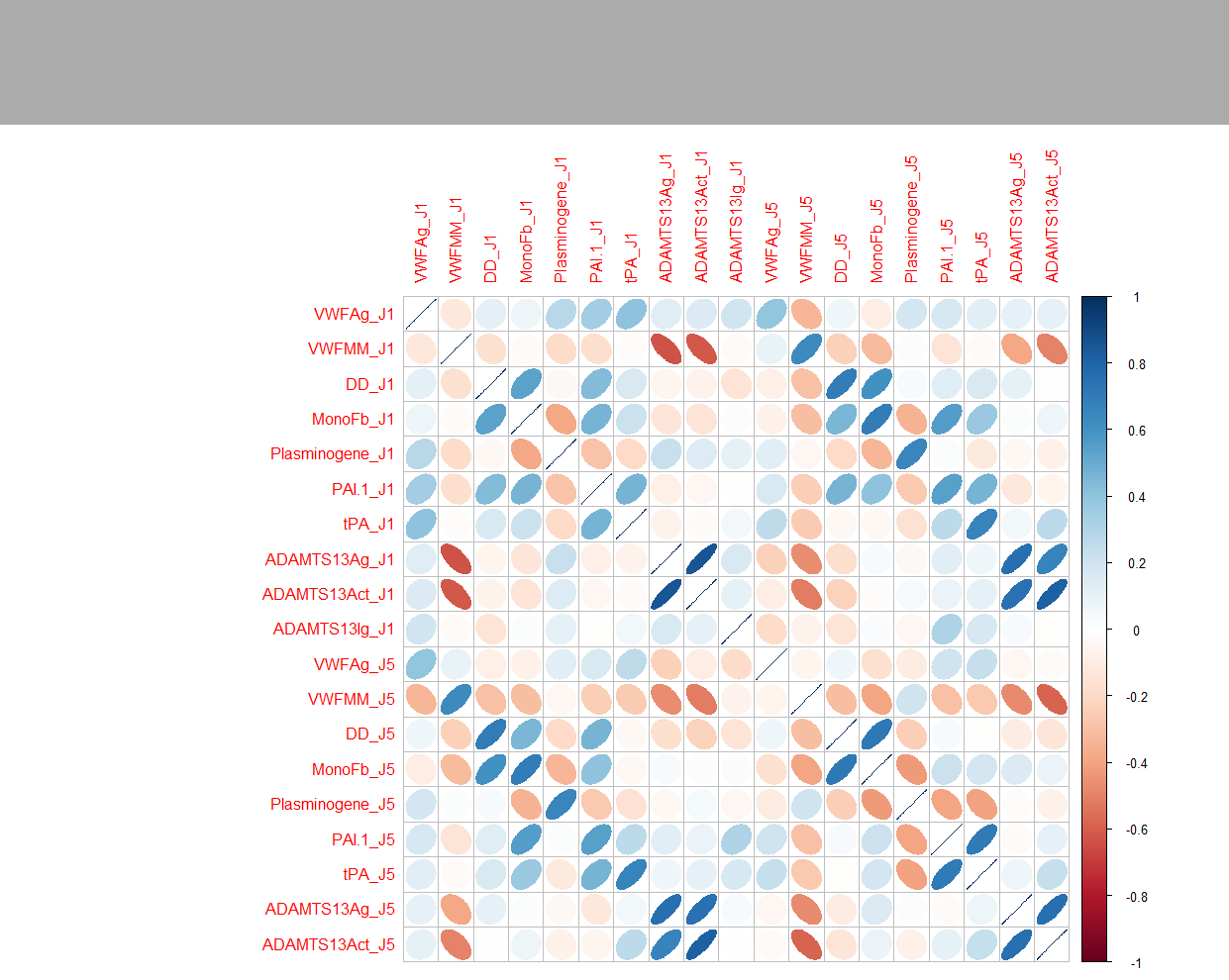

Supplement: S1 Fig — (TIF) [file pone.0251216.s003.tif]

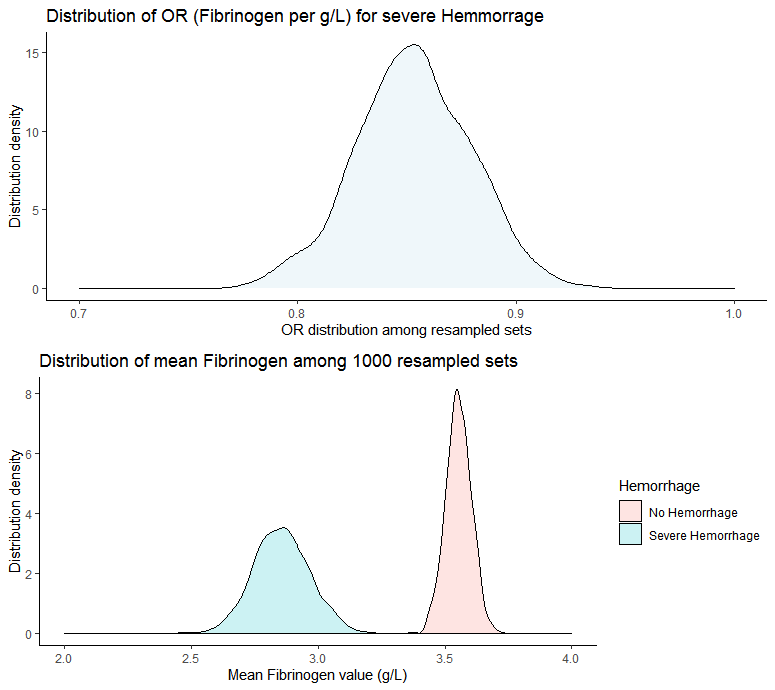

Supplement: S2 Fig — (TIF) [file pone.0251216.s004.tif]
